# Exploration of Oral Sodium Bicarbonate Acceptance and Adherence among Chronic Kidney Disease Patients with Metabolic Acidosis: A Multicentre Cross-Sectional Survey

**DOI:** 10.21315/mjms-09-2024-693

**Published:** 2025-02-28

**Authors:** Jaime Yoke May Chan, Farida Islahudin, Mohd Makmor-Bakry, Nurul Ain Mohd Tahir, Clare Hui Hong Tan

**Affiliations:** 1 Centre for Quality Management of Medicines, Faculty of Pharmacy, Universiti Kebangsaan Malaysia, Kuala Lumpur, Malaysia; 2 Pharmacy Department, Hospital Umum Sarawak, Ministry of Health, Kuching, Sarawak, Malaysia; 3 Faculty of Pharmacy, Universitas Airlangga, Surabaya, Indonesia; 4 Nephrology Department, Hospital Umum Sarawak, Ministry of Health, Kuching, Sarawak, Malaysia

**Keywords:** acceptance, adherence, chronic kidney disease, metabolic acidosis, sodium bicarbonate

## Abstract

**Background:**

Oral sodium bicarbonate is recommended for treating metabolic acidosis in chronic kidney disease (CKD). However, limited information exists on patient preferences between sodium bicarbonate tablets and powdered solutions. This study aimed to provide baseline data regarding the acceptance and adherence of patients with CKD to oral sodium bicarbonate therapy.

**Methods:**

This prospective multicentre cross-sectional study was conducted across five Malaysian government hospitals involving adult patients with pre-dialysis CKD. A questionnaire assessed demographics, clinical characteristics, bicarbonate treatment, and included the Medication Acceptance Questionnaire (MAQ) (convenience, taste, appearance, efficacy, and tolerability), along with an individual adherence assessment.

**Results:**

Among 203 patients analysed, the median age was 60 years (interquartile range [IQR], 16 years), and the majority were at stage 5 (*n* = 138, 68.0%). Sodium bicarbonate acceptance scores above 70% for all MAQ domains were significantly higher among tablet users than those of the powdered solution users, who only had scores above 70% for convenience, taste and tolerability domains. Tablet users were more adherent to treatment (88.9% vs. 70.9%, *p* < 0.014). A positive correlation was found between self-reported adherence and all five MAQ domain scores for the oral powdered sodium bicarbonate solution (convenience: *r_s_* = 0.223, *p* = 0.005; taste: *r_s_* = 0.223, *p* = 0.005; appearance: *r_s_* = 0.161, *p* = 0.043; efficacy: *r_s_* = 0.247, *p* = 0.002; tolerability: *r_s_* = 0.279, *p* < 0.001). For tablet users, significant positive correlations were observed between self-reported adherence and the convenience (*r_s_* = 0.413, *p* = 0.005), appearance (*r_s_* = 0.449, *p* = 0.002), and efficacy (*r_s_* = 0.355, *p* = 0.017) domains.

**Conclusion:**

Tablet formulation of sodium bicarbonate was associated with higher patient acceptance and adherence, potentially leading to improved long-term clinical outcomes.

## Introduction

Chronic kidney disease (CKD) is a persistent condition necessitating comprehensive therapeutic intervention aimed at impeding disease progression, managing symptoms, and mitigating associated complications. In the pre-dialysis phase, treatment strategies predominantly focus on decelerating CKD advancement towards end-stage renal disease (ESRD), alongside addressing concurrent comorbidities and common complications such as hyperphosphatemia, anaemia, or acidosis. Upon dialysis initiation, the therapeutic objectives shift, emphasising complication management and the imperative task of averting the substantial morbidity and mortality risks inherent in maintenance dialysis (1). Consequently, patients with CKD often contend with intricate medication protocols characterised by high pill volumes administered over prolonged periods (1). Furthermore, the progressive nature of CKD necessitates continuous adjustments to medication regimens. Medication adherence in CKD resembles a protracted endeavour akin to a marathon, given that the efficacy of treatment protocols significantly affects sustained acceptance and adherence to prescribed medications, with meticulous adherence to prescribed dosages for symptom management and disease control (2, 3).

Metabolic acidosis, a complication often overlooked in CKD management, poses a significant clinical challenge (4). Its reported prevalence ranges from 6% to 80%, with an escalating incidence correlating with kidney disease progression (5, 6). The multifaceted consequences of metabolic acidosis have been extensively documented (7, 8) despite the limited treatment options available. Although evidence suggests potential therapeutic benefits, including preservation of kidney function, improvement of muscle mass, and insulin resistance (9–12), effective treatment modalities remain sparse. Current management protocols recommend the initiation of oral alkali salts or an alkaline-rich diet when the serum bicarbonate levels falling below 22 mEq/L (13). Notably, such interventions demonstrate a significant elevation in bicarbonate levels compared with their untreated counterparts (14). In critical care settings, intravenous sodium bicarbonate is often used despite its limited efficacy, particularly with respect to survival benefits in patients with acute kidney injury (15).

Oral sodium bicarbonate is a prevalent choice for managing metabolic acidosis and is commonly available in tablet and powder formulations because of its recognised safety profile, efficacy, and cost-effectiveness (8). Within the realm of CKD treatment, oral sodium bicarbonate tablets have demonstrated notable advantages in achieving target bicarbonate levels and enhancing adherence (10, 11, 16). Standard dosages typically range from 325 mg to 650 mg, with the bicarbonate content of one 650 mg tablet approximately equivalent to one-eighth of a teaspoon of sodium bicarbonate (8). In Malaysia, the available dosage form for tablets is 650 mg, while the powders are packed at 5 g, 33 g or 100 g per packet, based on the practice in different healthcare institutions. The use of sodium bicarbonate powder, while offering flexibility in administration, can pose challenges related to palatability, particularly in its reconstituted solution form, owing to its mildly alkaline or bitter taste (8). Such palatability issues may contribute to reduced adherence to alkaline therapy, a phenomenon observed with other medications, such as phosphate binders (17, 18). Non-adherence to prescribed medications not only compromises patient health outcomes, thereby exacerbating disease progression, but also imposes a substantial financial burden on healthcare systems (19–21).

Given the growing body of evidence highlighting the significance of alkaline therapy in addressing metabolic acidosis, ensuring patient acceptance and adherence to such treatment regimens is paramount for achieving therapeutic efficacy. This study aimed to provide baseline data on medication acceptability and adherence in patients with CKD undergoing oral sodium bicarbonate therapy. By elucidating these factors, healthcare providers can make informed decisions regarding the selection of the most suitable pharmaceutical formulation, thereby optimising treatment outcomes and cost-effectiveness in the CKD patient cohort.

## Methods

### Study Design

This was a prospective, multicentre, cross-sectional study conducted at five Malaysian government hospitals with nephrology subspecialties via convenience sampling. Patients that were included in this study were Stage 3 to 5 CKD patients aged 18 years and above on alkali treatment for metabolic acidosis. Incomplete questionnaires were excluded from analysis. A researcher-assisted questionnaire was used to elicit information such as acceptability, adherence, and knowledge, while patients’ clinic cards were reviewed for laboratory investigation results and a list of medications. Eight appointed researchers underwent training before data collection to standardise the data collection process. The study was performed according to the STROBE guidelines.

### Sample Size

The sample size n was determined using the two-population proportion formula (22):


$$n=\frac{{\left\{z_{1-\frac{\alpha }{2}}\sqrt{2\bar{p}(1-\bar{p})}+z_{1-\beta }\sqrt{p_{1}(1-p_{1})+p_{2}(1-p_{2})}\right\}}^{2}}{{\left(p_{1}-p_{2}\right)}^{2}}$$

Thus, the minimum required sample size for this study was 22 patients per group with *Z* = 1.96 and the significance level was set at 0.05, and the power was 80%. Prior data indicate that the proportion of patients who preferred the tablet formulation was 71% and the proportion of those who preferred the liquid formulation was 29% (23). To account for a 50% dropout rate, a minimum sample size of 44 participants per group was targeted (24).

### Data Collection

Data were collected using a standardised data collection form divided into two sections. The first section consisted of demographic and clinical characteristics, including sex, age, ethnicity, education, current CKD staging, co-morbidities, and medication characteristics, including the treatment history of oral sodium bicarbonate and laboratory parameters such as serum bicarbonate, serum potassium, and serum creatinine levels at baseline prior to initiation of oral sodium bicarbonate and at the time of the study. The second section included the Medication Acceptability Questionnaire (MAQ) (25) and medication adherence assessment which were divided into two parts based on previous studies: the CKD Adherence Prediction Score (CAPS) (26) and individual medication adherence assessment (27).

The validated MAQ (25) covers five domains: convenience, taste, appearance, efficacy, and tolerability. The convenience domain included statements pertaining to the convenience of dosing frequency, amount of medication administered, ease of administration, lifestyle, and convenience of administration when not at home. The taste domain focuses on the overall taste, presence of aftertaste, and texture of the medication. The appearance domain examines issues such as vision, colour, smell, and touch. The efficacy domain elicited patient responses regarding the effectiveness of the medication in terms of symptom alleviation and speed of drug action. The tolerability domain refers to the patients’ overall being and not the specific side effects of the medication in question. Each item was scored on a 5-point Likert scale, with 1 indicating “strongly disagree” and 5 indicating “strongly agree”. The scores of the items in each of the five domains were summed to obtain the domain score. The tolerability domain was scored inverted, with 1 indicating “strongly agree” and 5 indicating “strongly disagree”, as the items in this domain were negatively phrased. Because each domain had a different number of items, the domain scores were reported as percentages. Higher scores indicate greater acceptability of the formulation for the domain under investigation (25).

The CAPS is a medication adherence prediction model developed to help identify patients with a higher risk of non-adherence in the CKD population based on individual adherence to medications. The CAPS was used in this study to predict the patients’ risk of non-adherence to their overall medications by assessing each individual medication, including oral sodium bicarbonate. The CAPS is scored based on four components: number of prescribed medications, number of co-morbidities, use of complementary and alternative medication (CAM), and medication knowledge score. Patients with CAPS of ≤ 3.5 has a higher risk of non-adherence and requires closer monitoring (26). Medication knowledge for each medication under the CAPS was assessed based on the patient’s understanding and knowledge of the drug dose (D), frequency (F), indication (I), and time of administration (T) [DFIT]. Correct answers were scored “1” whilst incorrect answers were scored “0”. The overall knowledge score was based on the total number of correct answers divided by the total number of questions × 100% (28). In addition, individual medication adherence assessment was carried out via patient interviews and calculated based on the patient’s self-reported number of missed doses in the past month using the following formula: [(prescribed doses-missed doses)/total prescribed dose] × 100%. A “missing” dose was considered when the patient recalled not taking the prescribed medication(s) compared with the prescribed medication(s) listed in the institutional medical records. If the percentage of consumption of the prescribed drugs was higher or equal to 80%, the patients were then considered adherent. For patients who used more than one medication, the proportion of use was calculated for each drug and averaged according to the number of medications used (27).

### Statistical Analysis

Statistical analyses were performed using IBM statistics software (version 29; IBM Corp., Armonk, NY, USA). Categorical variables were presented in frequencies and percentages, while continuous variables were presented as mean ± standard deviation (SD) or median and range. Chi-square or Fisher’s exact tests were used to test the relationship between the two groups of sodium bicarbonate respondents and their demographic and clinical characteristics. The Mann-Whitney U test was used to compare the MAQ domain scores between the tablet and solution patients. The results are displayed using the median score, interquartile range (IQR), z-value, and p-value. The relationship between self-reported adherence scores for oral sodium bicarbonate and the five MAQ domains scores in percentages was analysed using Spearman’s correlation (*r_s_*). The self-reported adherence score was reported as numerical values (score 0–100) and the MAQ domain scores were presented as numerical values (0–100%). The *r_s_* values apply to both positive and negative correlation with the following categories: 0.00–0.25 means “little correlation”, 0.26–0.49 means “low correlation”, 0.50–0.69 means “moderate correlation”, 0.70–0.89 means “high correlation” and 0.90–1.00 refers to “very high correlation” (29).

## Results

### Patient Characteristics

**Figure 1:**
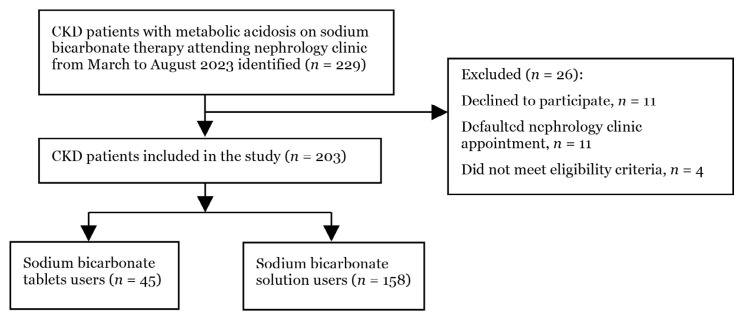
Study population selection flowchart

Two hundred and twenty-nine CKD patients with metabolic acidosis who were prescribed sodium bicarbonate therapy were identified, but only 203 were included in the analysis (Figure 1).

The median age of this study cohort of 203 patients was 60 years (IQR, 16 years), with 57.1% (*n* = 116) female and 39.9% (*n* = 81) Malays. The majority were stage 5 pre-dialysis CKD patients with an eGFR (glomerular filtration rate) of less than 15 ml/min/1.73 m^2^ (*n* = 138, 68.0%). The median number of medications prescribed was 8 (IQR: 3), and the number of medications ranged from 2 to 16. Most patients handled their own medications (*n* = 180, 88.7%) and denied the use of complementary and alternative medicine (*n* = 185, 92.5%). Two sodium bicarbonate formulations were identified, of which 77.8% (*n* = 158) were prescribed sodium bicarbonate powder that was reconstituted to solution and 45 patients (22.2%) were prescribed sodium bicarbonate tablets. Patients taking sodium bicarbonate tablets scored higher on overall medication knowledge (median score 100.0, IQR 5.9) than those taking the solution formulation (median score 93.8, IQR 13.1, *p* <0.001) (Table 1).

### Acceptance of Oral Sodium Bicarbonate

Each domain within the MAQ was summed and reported as a percentage (Table 2). Higher scores indicate greater acceptability of the formulation for the domain under investigation. The scores for all five MAQ domains were significantly higher in the tablet group than in the solution group (*p* < 0.05). The average scores for the convenience, taste, appearance, efficacy, and tolerability domains for the solution group ranged between 66.7% and 80.0%, whereas the average scores for similar domains for the tablet group ranged from 73.3% to 100.0%.

### Adherence to Oral Sodium Bicarbonate and Overall Medications

Self-reported adherence to oral sodium bicarbonate was higher in the tablet group than in the powdered solution group (88.9% vs. 70.9%, *p* = 0.014). Based on the CAPS, which addressed the prediction of the overall medication adherence of patients, there was no significant difference between the two formulations (both groups had a median CAPS of 5, IQR 2, *p* = 0.426) (Table 3).

### Relationship between Adherence and Acceptance of Oral Sodium Bicarbonate

There was a significant correlation between the self-reported adherence score for oral sodium bicarbonate and all five MAQ domains scores representing acceptance of oral sodium bicarbonate (Table 4). The observed correlation coefficients (*r_s_*) ranged from 0.255 to 0.307, suggesting a positive but weak correlation (29). When comparing the formulations separately, there was a significant correlation between the oral sodium bicarbonate solution and all five MAQ domain scores (convenience, *p* = 0.005; taste, *p* = 0.005; appearance, *p* = 0.043; efficacy, *p* = 0.002; and tolerability, *p* < 0.001). Significant correlations were also observed for oral sodium bicarbonate tablets in terms of convenience (*p* = 0.005), appearance (*p* = 0.002), and efficacy (*p* = 0.017). No significant correlation was demonstrated between the taste and tolerability domains and the self-reported adherence score for sodium bicarbonate tablets.

## Discussion

Our study demonstrated that those receiving sodium bicarbonate tablets exhibited higher scores across all five domains of the MAQ, particularly the convenience, taste, and appearance domains, indicating greater acceptability of the tablets than of the solution. These findings underscore the importance of considering patient preferences when selecting tablet and solution formulations for sodium bicarbonate therapy. Our findings are similar to those of previous studies comparing tablet and solution formulations of other medications (23, 30). For instance, a study comparing tablet and solution formulations of vitamin D revealed that 71% of participants favoured solid forms (tablets and capsules) over liquid forms (oily drops and alcoholic solutions) (23). Additionally, a study assessing responses to various formulations for the treatment of mild agitation reported a preference for tablets and orally dissolved tablets over liquid formulations (30).

Tablets generally offer advantages such as enhanced stability, longer shelf life, portability, ease of storage, accurate dosing, and the ability to mask the unpalatable taste of active ingredients or excipients, whereas solutions often present challenges such as shorter shelf life, difficulties in transport, and a greater likelihood of an unpalatable taste, despite facilitating faster digestion and absorption (31, 32). These observations are consistent with our study findings, in which participants in the solution group needed to prepare fresh solutions every two–four weeks based on facility instructions, whereas those provided with tablets typically benefited from longer expiry dates. Portability was highlighted in the convenience domain, with the majority indicating that tablets were more portable than solutions. Additionally, the median acceptance score for the taste domain was lower in the solution group than in the tablet group because the sodium bicarbonate solution tended to possess a slightly alkaline and bitter taste, potentially rendering it unpalatable.

Medication adherence among CKD patients presents a dynamic spectrum, with reported rates spanning from 3% to 88% across diverse assessment methodologies (1, 17, 33).

While various factors contribute to non-adherence, including fear of side effects, forgetfulness, cost, aversion to medication taste, and suboptimal communication with healthcare providers (1, 17, 33), emerging evidence highlights the importance of the medication form (23, 30, 32). Studies have consistently shown lower adherence to liquid medications than to tablets (23, 34). Our study supports this finding, demonstrating that patients with CKD are more likely to adhere to sodium bicarbonate therapy when prescribed in tablet form. The observed difference in adherence between the tablet and powdered solution groups underscores the need to consider formulation preferences when developing adherence strategies for sodium bicarbonate treatment. Despite this, the similarity in CAPS between the tablet and powdered solution groups in our study suggests that the medication form may have a minimal impact on the prediction of overall medication adherence in CKD patients. Future research should explore comprehensive interventions that address the multifaceted determinants to improve treatment outcomes in this population.

Measuring patients’ medication acceptance holds promise in predicting their behaviour towards treatment, encompassing both adherence and persistence (2). However, it is essential to recognise that medication acceptance does not necessarily equate to adherence, as acceptance reflects patients’ inclinations towards medication use, whereas adherence reflects their actual adherence to treatment. Our study contributes to this understanding by revealing a significant positive correlation between self-reported individual adherence scores and each domain of the MAQ for the solution group. Notably, medication-specific barriers such as palatability and side effects can undermine adherence in patients with chronic conditions (17). Our findings suggest that factors such as palatability, convenience, and side effects may contribute to a higher risk of non-adherence in both the solution and tablet groups. However, adherence to the prescribed sodium bicarbonate tablets appears to be unaffected by taste or tolerability issues. These insights underscore the complex interplay among medication acceptance, adherence behaviours, and formulation-specific factors, emphasising the need for tailored interventions to optimise treatment adherence and persistence in chronic disease management.

To the best of our knowledge, this is the first study to compare oral sodium bicarbonate solution and oral sodium bicarbonate tablets for the treatment of metabolic acidosis in a population with CKD, with findings that support the use of tablets. However, this study had several limitations. First, the study was constrained by the small sample size of patients receiving oral sodium bicarbonate for metabolic acidosis. Second, the study was conducted exclusively across five Ministry of Health hospitals, which potentially limits the generalisability of the findings. Third, clinical outcomes, such as the effectiveness of sodium bicarbonate for the correction of metabolic acidosis and the effect of sodium bicarbonate supplementation on CKD progression or CKD patients’ quality of life, were not examined.

Additionally, recall bias in self-adherence reporting poses a notable limitation, as some patients may have inaccurately recalled missed doses over the preceding month. Objective adherence measures, such as pill count, were not carried out because patients did not bring their balance stock of medications for their clinic follow-up appointments, and pharmacy refill data were not available for patients who collected their medications from other hospitals or healthcare facilities that were not study sites. Efforts to address these limitations, such as expanding the sample size and diversifying the study settings, are warranted to enhance the robustness and applicability of future research in this domain.

## Conclusion

Our study provides novel insights by demonstrating inferior patient acceptance of a sodium bicarbonate solution compared to tablets, suggesting a potential benefit in transitioning patients to the tablet formulation to enhance acceptability, adherence, and, ultimately, clinical outcomes. These findings could change the current practice in local settings, resulting in a switch from powdered solutions to tablets, provided that the tablet procuring process is streamlined. Involving patients in the decision-making process regarding their medication regimens may positively influence adherence and treatment outcomes. Future research should explore the budgetary implications associated with these two formulations to provide policymakers with valuable data to consider incorporating tablets into the national drug formulary. Such considerations are crucial for optimising treatment strategies and promoting equitable access to effective therapies within healthcare systems.

## Figures and Tables

**Table 1:** Demographics, clinical, and medication characteristics of the study population (*n* = 203)

Characteristics	Total on sodium bicarbonate therapy (*n* = 203)	Sodium bicarbonate solution (*n* = 158) *n* (%)	Sodium bicarbonate tablet (*n* = 45)	*p*-value^a^
Age in years [median, (IQR)]	60 (16)	61 (15)	57 (21)	0.131^b^

Gender				0.807
Male	87 (42.9)	67 (77.0)	20 (23.0)	
Female	116 (57.1)	91 (78.4)	25 (21.6)	

Ethnicity				0.762^c^
Malay	81 (39.9)	65 (80.2)	16 (19.8)	
Chinese	46 (22.7)	34 (73.9)	12 (26.1)	
Indian	1 (0.5)	1 (100.0)	0 (0)	
Others^d^	75 (36.9)	58 (77.3)	17 (22.7)	

Education level				0.439
Illiterate	19 (9.4)	15 (9.5)	4 (8.9)	
Primary	57 (28.1)	48 (30.4)	9 (20.0)	
Secondary	94 (4.3)	72 (45.6)	22 (48.9)	
Tertiary	33 (16.3)	23 (14.6)	10 (22.2)	

eGFR (ml/min/1.73m^2^) [median, (IQR)]	10.9 (10.7)	10.6 (10.3)	12.3 (13.9)	0.130^b^

CKD stage				0.187
Stage 3^e^	9 (4.4)	8 (5.1)	1 (2.2)	
Stage 4	56 (27.6)	39 (24.7)	17 (37.8)	
Stage 5	138 (68.0)	111 (70.3)	27 (60.0)	

Number of co-morbidities [median, (IQR)]	2 (1)	2 (1)	2(2)	0.027^b^

Type of co-morbidities				
Diabetes mellitus	124 (61.1)	102 (64.4)	22 (48.9)	0.057
Hypertension	164 (80.8)	133 (84.2)	31 (68.9)	0.022
Ischaemic heart disease and other cardiovascular diseases	23 (11.3)	20 (12.7)	3 (6.7)	0.263
Dyslipidemia	16 (7.9)	11 (7.0)	5 (11.1)	0.356^c^
Gout	37 (18.2)	32 (20.3)	5(11.1)	0.161
Anaemia	10 (4.9)	9 (5.7)	1 (2.2)	0.464^c^

Number of medications [median (IQR)]	8 (3)	8 (3)	8 (3)	0.623^b^

Medications handled by				0.558
Patient	180 (88.7)	139 (88.0)	41 (91.1)	
Patient’s family member	23 (11.3)	19 (12.0)	4 (8.9)	

Complementary and alternative medicine use	15 (7.4)	12 (7.7)	3 (6.7)	1.000^c^

Overall medication knowledge score^f^ [median (IQR)]	95.0 (12.5)	93.8 (13.1)	100.0 (5.9)	< 0.001^b^

Current dose of sodium bicarbonate prescribed (g/day) [median (IQR)]	2.0 (1.5)	2.0 (1.5)	1.3 (1.3)	0.140b

*Notes:*

a Analysis performed using Chi-square test with a significance level of *p* < 0.05;

b Analysis performed using Mann-Whitney test with a significance level of *p* < 0.05;

c Analysis performed using Fisher-exact test with a significance level of *p* < 0.05;

d Other ethnicities included Iban (*n* = 47), Bidayuh (*n* = 25), Kenyah (*n* = 1), Dusun (*n* = 1), and Melanau (*n* = 1);

e Stage 3a and 3b;

f DFIT score = medication dosage, frequency, indication and timing score

**Table 2:** Domain item scores (%) for both sodium bicarbonate formulations^a^

Domain	Number of items in each domain	Powdered solution group score [median (IQR)]	Tablet group score [median (IQR)]	*p*-value^b^
Convenience	5	80.0 (8.0)	100.0 (20.0)	< 0.001
Taste	3	66.7 (33.3)	80.0 (26.7)	< 0.001
Appearance	6	80.0 (13.3)	100.0 (20.0)	< 0.001
Efficacy	3	66.7 (20.0)	73.3 (20.0)	< 0.001
Tolerability (inverted)	2	80.0 (10.0)	90.0 (20.0)	0.014

Notes:

a Domain score was calculated and reported as a percentage of the total possible score to adjust for domains with different numbers of items;

b Analysis performed using Mann-Whitney test with a significance level of *p* < 0.05

**Table 3:** Adherence to oral sodium bicarbonate and overall medications

Characteristics	Total on sodium bicarbonate therapy (*n* = 203)	Sodium bicarbonate solution (*n* = 158) *n* (%)	Sodium bicarbonate tablet (*n* = 45)	*p*-value^a^
Self-reported adherence assessment				
Adherence^b^ to oral sodium bicarbonate	152 (74.9)	112 (70.9)	40 (88.9)	0.014

CKD Adherence Prediction Score (CAPS)				
Uses ≤ 7 prescribed medications	85 (41.9)	66 (41.8)	19 (42.2)	0.957
Has ≤ 3 co-morbidities	184 (90.6)	140 (88.6)	44 (97.8)	0.062
Does not use complementary alternative medicine (CAM)	188 (92.6)	146 (92.4)	42 (93.3)	1.000^c^
Medication knowledge score ≥ 80%	179 (88.2)	137 (86.7)	42 (93.3)	0.225
Total CAPS score, median (IQR)	5 (2)	5 (2)	5 (2)	0.426^d^

Notes:

a Analysis performed using chi-square test with a significance level of *p* < 0.05;

b Adherence score of ≥ 80% is considered adherent for each medication (27);

c Analysis performed using Fisher-exact test;

d Analysis performed using Mann-Whitney with a significance level of *p* < 0.05

**Table 4:** Spearman’s correlations (*r_s_*) between domain scores of acceptance and self-reported individual adherence score to oral sodium bicarbonate

Domain	Correlation coefficient, *r_s_* (*p*-value)		

	Self-reported individual adherence score to oral sodium bicarbonate		

	Overall (*n* = 203)	Solution (*n* = 158)	Tablet (*n* = 45)
Convenience	0.307 (*p* < 0.001)	0.223 (*p* = 0.005)	0.413 (*p* = 0.005)
Taste	0.265 (*p* < 0.001)	0.223 (*p* = 0.005)	0.253 (*p* = 0.093)
Appearance	0.255 (*p* < 0.001)	0.161 (*p* = 0.043)	0.449 (*p* = 0.002)
Efficacy	0.294 (*p* < 0.001)	0.247 (*p* = 0.002)	0.355 (*p* = 0.017)
Tolerability (inverted)	0.296 (*p* < 0.001)	0.279 (*p* < 0.001)	0.264 (*p* = 0.080)
